# Aging Well: Using Precision to Drive Down Costs and Increase Health Quality

**DOI:** 10.20900/agmr20190003

**Published:** 2019-06-05

**Authors:** Rhoda Au, Marina Ritchie, Spencer Hardy, Ting Fang Alvin Ang, Honghuang Lin

**Affiliations:** 1 Department of Neurology, Boston University School of Medicine, Boston, MA 02118, USA; 2 Framingham Heart Study, National Heart, Lung, and Blood Institute, Boston, MA 01702, USA; 3 Department of Epidemiology, Boston University School of Public Health, Boston, MA 02118, USA; 4 Department of Anatomy & Neurobiology, Boston University School of Medicine, Boston, MA 02118, USA; 5 Section of Computational Biomedicine, Department of Medicine, Boston University School of Medicine, Boston, MA 02118, USA

**Keywords:** digital biomarkers, precision health, aging

## Abstract

Efforts to provide patients with individualized treatments have led to tremendous breakthroughs in healthcare. However, a precision medicine approach alone will not offset the rapid increase in prevalence and burden of chronic non-communicable illnesses that is continuing to pervade the world’s aging population. With rapid advances in technology, it is now possible to collect digital metrics to assess, monitor and detect chronic disease indicators, much earlier in the disease course, potentially redefining what was previously considered asymptomatic to pre-symptomatic. Data science and artificial intelligence can drive the discovery of digital biomarkers before the emergence of overt clinical symptoms, thereby transforming the current healthcare approach from one centered on precision medicine to a more comprehensive focus on precision health, and by doing so enable the possibility of preventing disease altogether. Presented herein are the challenges to the current healthcare model and the proposition of first steps for reversing the prevailing intractable trend of rising healthcare costs and poorer health quality.

## BACKGROUND

Age structures worldwide are experiencing an unprecedented shift where people over the age of 60 are expected to outnumber children between the ages of 0–9 by the year 2030 [1]. Data from World Population Prospects: the 2017 Revision, further project that the population of 962 million people over age 60 in 2017 will double to 2.1 billion by 2050 [2]. Increased longevity and decline in fertility rates fuel the rapidly aging population across the world. Although the rate of disproportionate growth is most advanced in Europe and North America, where people over the age of 60 account for 35% and 25% of the population respectively, other continents are joining the ranks at an accelerated pace [1]. This global change has shifted the leading cause of death worldwide to chronic non-communicable diseases highly concentrated in older ages. Of all the non-communicable diseases, cardiovascular diseases (CVD), cancers, diabetes and chronic obstructive pulmonary diseases (COPD) are the four dominant chronic conditions that pervade the world’s aging population in all socioeconomic classes [3,4]. Brain related disabilities also sharply correlate with increasing age. With much of the global burden attributed to chronic conditions that diminish wellbeing and one’s quality of life, it perpetuates a new public health challenge of “ageing well” [5]. Health is a prominent component of aging well. It is considered as “a state of an individual characterized by the core features of physiological, cognitive, physical and reproductive function, and a lack of disease” [6].

The high prevalence and burden of chronic illnesses in this aging demographic also imposes a substantial economic toll, which includes direct costs such as medical expenses and income losses. As an attempt to address chronic disease concerns, high healthcare costs have also been expended on research and education. Altogether, it was estimated that approximately 3.8 trillion global healthcare dollars (in U.S. dollars) were spent toward the four major diseases in 2010, where $2.1 trillion was spent on chronic respiratory disease, $863 billion on cardiovascular disease, $500 billion on diabetes and $290 billion on new cancer cases [7]. Assuming that the number of people affected by these disorders will continue to increase, simulations anticipate a cumulative loss of $47 trillion between 2011 and 2030, including the costs of mental health conditions that often accompany chronic diseases. This economic demand is a major concern in the U.S., as the country surpassed every other nation as the highest per capita earning and spending on healthcare [8]. Predictably, three quarters of U.S. healthcare spending is allotted to treating chronic diseases [9]. Yet, even with the large distribution of money for healthcare, the U.S. is ranked 37th in overall health system performance, which demonstrates the complexity of this challenge [10].

With advances in healthcare however, survivor statistics on all forms of cancer have and continues to strikingly improve. The U.S. experienced a 26% decrease in cancer mortality between 1991 and 2015, saving approximately 2,378,600 deaths [11]. Remarkable declines in mortality rates can also be seen in other chronic conditions. Between 2006 and 2016, number of deaths caused by coronary heart disease declined by 14.6% [12]. In particular, the dropping death rate in cerebrovascular disease has been celebrated as one of the top ten great public health achievements, falling from the third leading cause of death to the fourth [13]. The most prominent breakthrough, however, has been the conversion of Human Immunodeficiency Virus (H.I.V) infection from inevitable mortality to a manageable chronic condition. With the development of antiretroviral therapies, many initiatives have improved the prognosis of the disease, saving millions of lives around the world [14].

## PRECISION MEDICINE

Advances in technology and increased accessibility to patient data have given rise to the concept of precision medicine as a key movement to modify the daunting trajectory of global healthcare. By using a combination of genetic, behavioral and environmental indices, the approach identifies unique disease risks and strategically tailors therapeutic interventions for each patient [15]. The advantages and success of precision medicine has been highlighted in cancer, where the same disease can behave differently by patient throughout the clinical course of the illness [16]. Personalized therapies have already produced marked improvements in patients’ chance of survival, especially when the cancer is driven by genetic underpinnings. A clinical example of success in oncology includes the emergence of targeted therapies that intervene the growth and spread of cancer cells by depending on molecular and genomic profiles [17]. Identification of specific mutations and origins of cancer facilitate the application of differentiated therapies for different cancer subtypes. Directly targeting the source with greater precision reduces adverse symptoms often associated with conventional chemotherapy that act on rapidly dividing cells whether they are malignant or not [18,19]. Although this illustrates how precision medicine bring promise of “the right treatment for the right person at the right time”, this paradigm perpetuates a dichotomy of high health care costs and low health quality, as the majority of diseases are chronic. Thus, an emphasis solely on precision medicine ties solutions that are responsive to the rapid evolution of epidemics but does not sufficiently identify the root cause of the problem. To break the seemingly irreversible trend for ever increasing healthcare costs requires rethinking current approaches for addressing chronic disease risk and management.

## PREVENTION

Public health serves to reorient from treatment to prevention and efforts to do so are increasing. The first step of prevention begins with health promotion and identification of risk factors. The World Health Organization (WHO) predicts that by eliminating modifiable exposures, 80% of heart disease, stroke and diabetes can potentially be prevented [20]. A more recent study suggests that this is an underestimation; 10 modifiable risk factors accounted for 90% of cerebrovascular disease alone [21]. Similarly, researchers have also attributed 42% of all cancers to modifiable risk factors [22]. Looking more closely at common predictors, a study investigating the impact of 7 known CVD risk factors on 9 chosen chronic diseases (diabetes, hypertension, high cholesterol, asthma, arthritis, CVD, COPD, cognitive impairment and chronic kidney disease) revealed that smoking and obesity are individually large contributors, and together impact all 9 outcomes [23]. Extensive literature point to the same suggestion that as obesity rates rise, so do the prevalence and incidence of chronic conditions [24,25]. These findings encourage further honing of risk knowledge on an individual and population level to determine what early detection and health promotion efforts might be most effective to foster. While more studies are needed to ascertain causality, these results underscore the immense potential of risk factors for disease preclusion. This is true at all disease stages, in which the objectives include slowing onset, mitigating progression, or averting disease altogether.

Despite exhaustive literatures supporting the power of prevention, hurdles in implementation have limited the vision from transforming to reality. The overarching challenge stems from the different priorities and deficient collaborative commitments of key stakeholders in healthcare including individuals, health professionals, corporate sectors and government [26]. In addition to competing priorities, funding restrictions for prevention resources are also reported as a leading challenge [27]. On a narrower scope, healthcare experts also face ethical challenges that accompany aggressive preventative approaches [28]. However, as appealing as frequent checkups may sound, benign symptoms can sometimes appear as consequential diseases during screening, which often lead to over diagnosis and unnecessary treatment [28]. These obstacles, together with the current lack of framework at different levels of operation, impede the deployment of a range of prevention strategies.

Nevertheless, favorable outcomes follow when preventative schemes are successfully executed. As an example, smoking cessation initiatives have been pivotal in reducing morbidity in many countries. This is clearly reflected in the wide variability of smoking incidences by nation [29]. A meta-analysis reviewing the effectiveness of health promotion methods articulate the benefits of strategies that reach the individual, community and socio-political establishments through different channels of advocacy and campaign [30]. New York City’s approach to reducing sugary drink consumption serves as a case study of this tactic. Using smoking cessation campaigns as a blueprint, the New York City (NYC) Department of Health and Mental Hygiene (DOHMH) implemented a mass media educational campaign that exposed health consequences of sugary drink consumption [31]. This approach was complemented with institutional and policy change pitches, including a cap on sugary drink serving sizes, posting calorie counts on menus in food service establishments, instituting nutritional standards in facilities, *etc*. While more drastic approaches like excise tax and restricted access to Supplemental Nutrition Assistance Program (SNAP) benefits did not pass, the process itself provided an opportunity to re-emphasize the campaign’s central message. Over the course of 7 years, these initiatives accomplished a 35% decrease for NYC adults and 27% for public high school students in consuming one or more sugary drinks a day [31].

## PRECISION HEALTH

Advancements in technology is further pushing the boundaries of health reform and shifting the emphasis on precision medicine to precision health. As the concept of risk factors reveal, chronic diseases have an insidious onset and progression, which strongly encourage prognosis before the presentation of detectable clinical symptoms. This is well documented in studies that show associations between early-midlife risk and later life impact across different chronic disease states. A longitudinal study assessing successful aging reported that in addition to biological factors, not smoking, diet and exercise were linked to favorable outcomes free of major diseases and high functioning [32]. A study by Yaffe *et al.* corroborated these findings by demonstrating how early to midlife cumulative exposure to cardiovascular risk factors including higher systolic and diastolic blood pressures and fasting blood glucose are associated with worse outcomes on neuropsychological tests [33]. The 2-year Finnish geriatric intervention study to prevent cognitive impairment and disability (FINGER) demonstrated that lifestyle interventions were effective in reducing risk for cognitive decline, but additional studies did not confirm these findings [34].

Thus, while the overall compendium of research suggests modifiable risk factors are central to chronic disease prevention, effective intervention methods are still elusive. Nonetheless, spurred by these promising findings, another ongoing national momentum is the implementation of setting-based approaches, as many companies now have increased healthcare benefits to include coverage of wellness programs and services. Insurance companies and provisions through The Affordable Care Act (ACA) not only facilitate productivity in the work place but also serve to minimize the economic burden of chronic diseases [35]. Integrated solutions that target common underlying risk factors can now be delivered through models like service as a software (SaaS), as the industrial landscape digitizes their infrastructure.

The challenge remains on how to maximize chronic disease prevention opportunities with interventional methods that not only replicate the success of the FINGER study, but potentially enhance and expand it. Assessment and monitoring largely relies on sporadic and often self-reported methods. Digital technology provides a tool in which to more accurately detect, monitor and assess critical metrics and intervene and to do so beyond the walls of the clinic. Remote monitoring can identify symptom changes well below current thresholds that rely on less precise instrumentation. The granularity of data captured from digital technology will document initial signs of change well within the realm of normal, leading to redefinition of “asymptomatic” to “presymptomatic” and likely identify sensitivity periods in which interventions will be most effective.

## DIGITAL TECHNOLOGY AS A TOOL TO FACILITATE PRECISION HEALTH

Emergence of diverse Internet of Things (IoT) technologies such as wearable sensors, smartphone applications and smart home devices have also given rise to the concept of digital biomarkers. The idea is to enhance current gold standard tests and develop digital surrogates to traditional biomarkers. By leveraging these technologies to capture diverse metrics of health and human performance, it generates a wealth of data at a level of precision that accurately and objectively elucidates the heterogeneity of health parameters. Importantly, these methods are deployable at scale and at lower costs. With a surge of interest in digital biomarkers from chief health delegates like the Food and Drug Administration (FDA) and National Institute of Health (NIH), innovative action plans are beginning to emerge [36,37]. For instance, in 2018, the National Institute on Aging (NIA) has launched funding opportunity announcements to the Alzheimer’s Disease Research Centers to create biomarker cores including digital. The Alzheimer Drug Discovery Foundation also created a digital biomarker accelerator program [38,39].

To fully capitalize on its healthcare potential, digital technologies need to be validated against current clinical gold standard assessments and established biomarkers such as those from blood samples and imaging. Physical functioning is a behavioral measure widely used to assess risk for a range of chronic conditions. Balance and gait parameters have been at the forefront of digital biomarker research, as technologies like accelerometers and video recordings can easily add a digital layer on top of accepted protocols like the Timed Up and Go (TUG) test and the 6 Minute Walking Test (6MWT). Gait analysis has been implemented in clinic-based tests as a risk marker of diseases including COPD [40], heart diseases [41], neurological diseases [42,43] and diabetes [44]. In a similar way, conventional cognitive assessments can also be supplemented with digital technology. Long used written tests like the Clock Drawing Test have been augmented with a digitizing ballpoint pen while preserving the validity and reliability of the assessment as a screening tool for cognitive impairment and dementia [45,46]. As the pen collects raw data points that are also time-stamped, the full sequence of performance can be captured without changing the user experience. Similarly, a study using audio recordings of neuropsychological tests in the Framingham Heart Study have shown that certain voice features like decreased pitch and jitter, shorter segments of speech, and responses phrased as questions were positively associated with cognitive impairment [47]. Together, these studies have shown that with digital biomarkers and machine learning techniques, predictable models can display patterns that distinguish one disease from another and uncover preclinical patterns of disease onset. Further, of immediate clinical relevance is that these digital assessments coupled with standard in-clinic assessments provide a more holistic and detailed view of a person’s health that can lead to actionable interventions at a much earlier point than is typical of current healthcare practice. Such big data have already been considered for decision making during clinical practice. Until recently, continuous vital sign monitoring was limited to intensive care units (ICU) and became intermittent once transferred to the general wards. However, a systematic review by Downey *et al.* demonstrated that continuous monitoring past ICU care using minimally intrusive wireless technologies not only improved patient outcome but was also cost-effective [48]. Machine learning and analytics applications can also synthesize big data and improve data mining processes to extract relevant and insightful trends in a clinical context [49]. While clinical integration of remote IoT technology is still hindered by the lack of validation and potential issues of security and privacy, contributions of high-volume digital data can be pivotal to improve operations in health systems [50]. A significant barrier to clinical application is the fact that primary care providers and other healthcare professionals may not yet be fluent with these novel indices. Substantial efforts are still required to provide appropriate training throughout an adoption period.

The increasing use of smartphones and “smart home” applications have the potential to break down barriers to health assessment and monitoring of virtually anyone, regardless of their education, race/ethnicity, income level, geographical location or language. While the current digital divide cannot be overlooked, the increasing penetration of mobile phones, particularly within emerging economies, portend a future for the democratization of healthcare [51]. Technology makes possible shifting research and best practices from largely Caucasian-centric populations to a more comprehensively inclusive global one. In collaboration with medical research centers, technology companies worldwide are developing a plethora of health applications from surveys and functional tasks to educational information for various diseases. *mPower*, for example, is an application designed by joint efforts of Sage Bionetworks and the University of Rochester Medical Center (URMC) to allow researchers to monitor and track symptoms of Parkinson’s Disease (PD) [52]. By analyzing data on activities of daily living, modulating factors of various PD symptoms can potentially be discovered from which current treatment and other interventional strategies can be aligned and new ones can be developed [53]. Clinical trials have also benefited from the accessibility of mobile health (mHealth) technologies. In addition to accelerating recruitment, using these technologies to identify early signs of disease can help to pinpoint the correct patients to be included into clinical trials. Once enrolled, digital technology monitoring can also replace or augment subjective self-report measure with more objective health metrics. Given the granularity of these measures, researchers can more easily pick up on red flags that indicate when subjects should terminate/continue their participation. It should also be noted that trial costs spent on health devices can be offset by maximizing data collection through participants’ own smartphones [54]. Multi-site studies in particular experience collateral benefits as mHealth technology standardizes the data collection method, thereby improving the reliability of data [55]. While the majority of current technologies still require active engagement from the user standpoint, smartphone/tablet applications provide new opportunities to collect data passively and generate real-time feedback. As smartphone upgrades include more and better sensors, they will yield higher-dimensional data from everyday phone-user interface. The emergence of 5G networks will enhance the discovery of novel digital biomarkers of chronic conditions exponentially. Further, digitization has also been fueling the shift of data collection to being increasingly more person-centric, which will ultimately make the healthcare ecosystem not only more efficient but also more accountable.

While the conceptual vision of digital biomarkers is exciting, the operationalization of techniques and a strategic framework have yet to be made concrete. Delineation of regulatory processes is also needed to make digital biomarkers a reality. One key principle in making sustainable progress is the idea of open access data sharing. Consistent with the NIH data sharing repositories like NIA Accelerated Medicines Partnership-Alzheimer’s Disease Knowledge Portal or the Health and Retirement Study, it is essential to make data accessible to the research community [56,57]. Inviting the community at large to mine the data and build predictive algorithms will produce a multitude of translational models, well beyond the capacity of any individual or single team of investigators and severely compress the timeline of discovery. Exclusive data archives should be challenged, as reduction in repetition through data transparency is indispensable to rapid resolution of the much more pressing and escalating threat of chronic diseases. Simultaneously, advanced analytic capabilities by data science experts and the evolving artificial intelligence (AI) community will fuel scientific invention across all diseases at an exponential rate. With enhancements in technological tools and datasets becoming more interconnected, discussions on the risks of re-identification have surfaced [58]. While this poses a potential challenge, powerful anonymization tools and protection technology are concurrently evolving to minimize the risk. Along with developing advanced de-identification tools, a formalized protection framework should be placed on all open access data [59]. As such, confidentiality and patient privacy should always be prioritized and at the forefront of healthcare innovation.

## CONCLUSIONS

Technology is reshaping the way we think of health and will transform current medical and public health practice. The economic impact will be substantive, providing a roadmap for reining in healthcare expenditures and improving health status. The therapeutic arena is already experiencing affirmative outcomes through improved technology-based interventions, with accelerated expansion made possible through the emergence of prognostic digital biomarkers. Capturing sensitive disease-related measures will facilitate early action that could mitigate or modify the course of disease whose heterogeneous expression have made effective treatments to date fleeting. Clinical studies and animal models should be further validated so that a wider range of interventions can be available for individuals to apply a combinatory approach for health promotion [60]. With greater personalization of healthcare in all stages of the life course, the public health perspective is shifting from precision medicine to precision health. Furthermore, complementary to the medical model is an emergent new business ecosystem comprised of tens of thousands if not more new technology companies centered on providing the right solution to the right person at the right time. Given the global pandemic of chronic diseases, the challenge now is to bring together these key stakeholders to constrict the timeline of reaching the precision health objective. Interdisciplinary contributions that are harmonized and broadly shared are central to finding real solutions that will drive down healthcare costs and increase health quality.
